# Understanding the menopausal experiences of women with intellectual disabilities: A scoping review

**DOI:** 10.1177/17446295231182246

**Published:** 2023-06-15

**Authors:** Katie Moore, Mary Reidy, Sinead Foran

**Affiliations:** Department of Nursing and Health Care, 8807 South East Technological University, Waterford, Ireland; Department of Nursing and Health Care, 8807 South East Technological University, Waterford, Ireland; School of School of Nursing, Psychotherapy and Community Health, 8818 Dublin City University, Dublin, Ireland

**Keywords:** intellectual disabilities, menopause, reproductive health, aging, scoping review

## Abstract

During the process of ageing, women experience important hormonal, endocrine and biological changes. Menopause is a natural phenomenon in female development, during which women’s ovarian function shifts from a reproductive to a non-reproductive state. The experience of menopause is unique for every woman, including women with intellectual disabilities. Globally, the available literature on women with intellectual disabilities and menopause focuses on providing medical insights into onset and symptoms and little attention has been paid to documenting how menopause affects women themselves. This represents a significant gap in understanding how women understand this change in life and has been a key justification for the need for this research. This scoping review aims to consider published studies capturing the perceptions, experiences and attitudes of women with intellectual disabilities and their caregivers as they transition through the menopause.

## Background

During the process of ageing, women experience important hormonal, endocrine and biological changes. Menopause is a natural phenomenon in female development, during which women’s ovarian function shifts from a reproductive to a non-reproductive state (Almeida and Greguol, 2015; Ilankoon et al., 2021) caused by a decrease in the hormones, oestrogen and progesterone. The term ‘menopausal transition’ refers to the period leading up to menopause and is also known as ‘peri-menopause’. During this stage, menstruation becomes irregular. Women are considered to be in this phase of transition when their last normal menstrual bleed occurred within the previous 3–12 months (Stanzel et al., 2022). Natural menopause is said to have occurred when a woman reports an absence of menstruation for 12 consecutive months (Hoga et al., 2015). Post-menopause is the stage following the last spontaneous bleed and begins 12 months after a woman’s final menstrual cycle (Stanzel et al., 2022). Throughout this review, the term menopause is used to refer to all stages of menopause including peri-menopause and post-menopause unless it is explicitly referenced.

Discussions about menopause are becoming mainstream and have gained traction with global media coverage. Notwithstanding this progress, women with intellectual disability have been unrepresented in the discourse. The literature on women with intellectual disabilities and menopause focuses on medical insights with little attention paid to understanding the impacts of menopause on women’s psychological, physical and emotional wellbeing. The experience of menopause is unique for every woman, including women with intellectual disabilities. While some women experience few symptoms, others may encounter significant physical and emotional effects that require medical treatment. Severe menopausal symptoms can have a profound impact on women’s overall wellbeing, personal and social interactions, and quality of life (Kaunitz and Manson, 2015; Taebi et al., 2018; Vandenakker and Glass, 2001).

Emerging epidemiological studies of menopause provide reliable evidence relating to the incidence, prevalence, and severity of several menopausal symptoms and indicate that there are subsets of women who are more or less vulnerable to particular symptoms or sets of symptoms (Santoro et al., 2015). Although there is a paucity of literature documenting the menopausal symptoms of women with intellectual disabilities, it is suggested that women in this population experience the same range of symptoms during and after menopause as women in the general population (Martin et al., 2001; Trueland, 2023), yet for these women symptoms of menopause can be additionally complex particularly in cases where there are enduring health issues.

Vasomotor related symptoms are the most widely reported signs of menopause, experienced predominantly during the peri-menopause stage (Arnot et al., 2021). These symptoms include night sweats, chills, anxiety, insomnia, heart palpitations and in particular hot flushes (Kalarhoudi et al., 2011; Kaunitz and Manson, 2015). Hot flushes are the most common complication of menopause, with a reported prevalence rate of 20-80% (Chiang et al., 2019; Hardy et al., 2018; Samami et al., 2022). Other symptoms include nervous system related issues such as weight gain, muscular and skeletal issues, cardiovascular diseases (Erbil, 2018; Monteleone et al., 2018), headaches and mood swings (Kalarhoudi et al., 2011). Alongside the hormonal and biological symptoms, there are other psychological impacts of menopause. Milner et al., 1997 describe psychological symptoms such as depression, anxiety, panic attacks and changes in libido (Milner et al., 1997). These symptoms may not be directly attributable to the change in hormone production that occurs during menopause, but may arise as a result of a combination of other affective, contextual, and lifestyle factors that impact women during this time (Elavsky and Mcauley, 2009; Lindh-Astrand et al., 2007). Symptoms may be further influenced by individual personality traits, such as how a woman perceives, intreprets and copes with menopausal symptoms (Jalava-broman et al., 2020).

Many women with intellectual disabilities face ongoing challenges in the promotion of their health and wellbeing. In this population, the prevention of secondary disabling conditions, such as osteoporosis, fatigue, and skin breakdown can be compounded by the onset of menopause in midlife (Dormire, 2007). Further, there is limited literature documenting the impact of the decreases in hormones after menopause on the cognitive capacity (Walsh et al., 2001), seizure patterns (Brown and Murphy, 1999) or psychological health of menopausal women with intellectual disabilities. It is suggested that the psychological impact of the symptoms of menopause may be exacerbated for some women by a lack of understanding of what is happening to their bodies and where women live in residential homes having relatively little privacy may cause additional stress (Mccarthy and Millard, 2003).

Existing literature suggests that the onset of menopause occurs earlier in women with intellectual disabilities than for women in the general population, and even earlier in women with Down Syndrome (Carr and Hollins, 1995; Cosgrave et al., 1999; Schupf et al., 2003). While the menopausal experiences of women with and without intellectual disabilities are understood to be similar on a physiological level, there is little acknowledgement of the lived experiences of women with intellectual disabilities, and their perceptions about the transition into menopause are largely underrepresented in the data. Moreover, much of the available research, as demonstrated in this review, focuses on the perceptions of formal and informal caregivers of the experiences of the women with intellectual disabilities in their care. This represents a significant gap in understanding how women understand this change in life and has been a key justification for the need for this research. This scoping review aims to consider published studies capturing the perceptions, experiences and attitudes of women with intellectual disabilities concerning menopause and their perceived experiences as observed by their caregivers.

Issues of disability and menopause have featured in conceptual and review papers, particularly over the previous two decades, and inspired empirical research in this area in the early 2000’s. However, as discussions of menopause re-emerging in mainstream narratives and given that the research area is still in its relative infancy, it was important to carry out an exploratory review of the existing literature to aggregate the evidence base, assess the quality of the available studies and highlighting the gaps that require further investigation (Arksey and O’Malley, 2005). In the disciplines of health and social sciences, the use of scoping reviews has increased in popularity as this approach is recognised as an appropriate method of inquiry for informing new research in these areas (Pham et al., 2014; Tricco et al., 2016). Scoping review methods enable researchers to examine the existing literature, record what and how previous research has been conducted, identify key factors related to the central concepts and analyse the gaps for further research (Munn et al., 2018; Peters et al., 2020). Intellectual disability in itself is broad, complex and can be multifaceted in terms of its epidemiology, how it is characterised and how it is experienced on an individual level. As such, a scoping review is considered an appropriate methodology for examining the different types of evidence related to intellectual disability and menopause.

While the employment of scoping reviews has become more popular across the disciplines, so too have the criticisms of the approach with some authors highlighting weaknesses in it's use for synthesising evidence (Davis et al., 2009; Tricco et al., 2016). A scoping review is an method for providing a comprehensive overview of evidence , rather than a type of systematic review which seeks to synthesis qualitative or quantitative data (Munn et al., 2018) and as such, scoping reviews do not necessitate critical appraisal (Lockwood et al., 2019; Peters et al., 2020) The seminal framework for conducting scoping reviews was developed by Arksey and O’Malley, 2005, however, since it’s conception, methodological challenges in this approach with regard to the analysis of data have been highlighted. In response to this, an extension of the framework was proposed by Levac et al., 2010 and updated guidance from the JBI and the Preferred Reporting Items for Systematic Reviews extension for Scoping Reviews (PRISMA-ScR) explicitly reference the need for increased rigour and transparency in the conduct of scoping reviews (Peters et al., 2020).

This paper aims to review literature published over the last two decades which examined the experiences of menopausal transition (including peri- and post-menopause) for women with intellectual disabilities. The objectives of the present review were to examine the range and nature of the published academic research and summarise the research findings to inform future policy, practice and guidelines for focused management of the health care of menopausal women with intellectual disabilities.

## Methods

The methods employed for this review were developed in line with guidance set forth by Arksey and O’Malley, 2005 and Levac et al., 2010. The methodological approach outlines five iterative stages for conducting a scoping review: (1) identifying the research question; (2) identifying relevant studies; (3) selecting studies; (4) charting the data; (5) collating, summarising and reporting the results. The review was conducted according to the PRISMA-ScR framework (Tricco et al., 2018) to structure the results to ensure the rigour, trustworthiness and methodological transparency of the findings.

### Stage 1: Identifying the research question

The overarching research question: What are the menopausal experiences of women with intellectual disabilities?

To provide more specific direction for the research and to further break down particular aspects of the overarching research question three sub-questions will be explored, namely,

1. What knowledge do women with intellectual disabilities have about menopause?
2. Where do women with intellectual disabilities get their information about menopause?
3. What interventions are used with women with intellectual disabilities who have symptoms of menopause?

### Stage 2: Identifying the relevant studies

A comprehensive search was conducted by searching and reviewing the literature from multiple sources (Arksey and O’Malley, 2005; Levac et al., 2010). Published literature in several medical, health-related, social, psychological and social science databases were systematically examined. Databases were selected for their likelihood to contain publications relevant to the review and these included Ebsco (including CINAHL and MEDLINE), Scopus, PubMed and ScienceDirect. Reference lists of included studies were examined to identify additional references of relevance.

Search terms were compiled based on keywords from the literature and in consultation with the institutions reference librarian where final terms and search strings were iteratively developed. The databases searched for studies published before March 2022 using Medical Subject Headings (MeSH) and subject index terms including the keywords, (menopause* OR menopausal* OR Perimenopause*) AND (intellectual disability* OR learning disability*) AND (experience* or perceptions* or attitudes*). The results were crossed checked to ensure seminal academic work in the area were captured in the results.

### Stage 3: Study selection

To ensure the appropriateness of the key search terms, the keywords and selected databases a pilot exercise of the search strategy was conducted. The researcher collaborated with an experienced librarian to develop the search strategy which was adapted and revised as necessary. A two-phase review process was then carried out. The first phase included the screening of the title and abstract of all identified citations against the pre-established eligibility criteria. The screening of titles and abstracts was guided by the PEO framework (Table 1) and relevant articles progressed to the second phase. One researcher conducted the initial screening of the identified articles, which were then cross-checked and validated by two reviewers. In phase two, the full-text publications of titles identified in phase one were screened relative to the inclusion and exclusion criteria. All remaining citations were exported to EndNote 20.1 reference manager where duplicate articles were be removed.

**Table 1.** PEO framework for study eligibility.

Criteria	Determinants
Population	Women with intellectual disabilities
	Formal and informal caregivers of women with intellectual disabilities
Exposure	Peri-menopause
	Menopause
	Post-menopause
Outcomes	Experiences of menopause
	Knowledge of menopause
	Treatment of menopausal symptoms

Studies were included in the review if they meet the following set of inclusion criteria:

• Reported on menopausal experiences of women with intellectual disabilities
• Included women with intellectual disabilities or their formal/informal caregivers
• Published in English
• Published between 2000 and 2022
• Included qualitative, quantitative or mixed methods methodologies or were reviews or commentaries.

**Figure 1.**
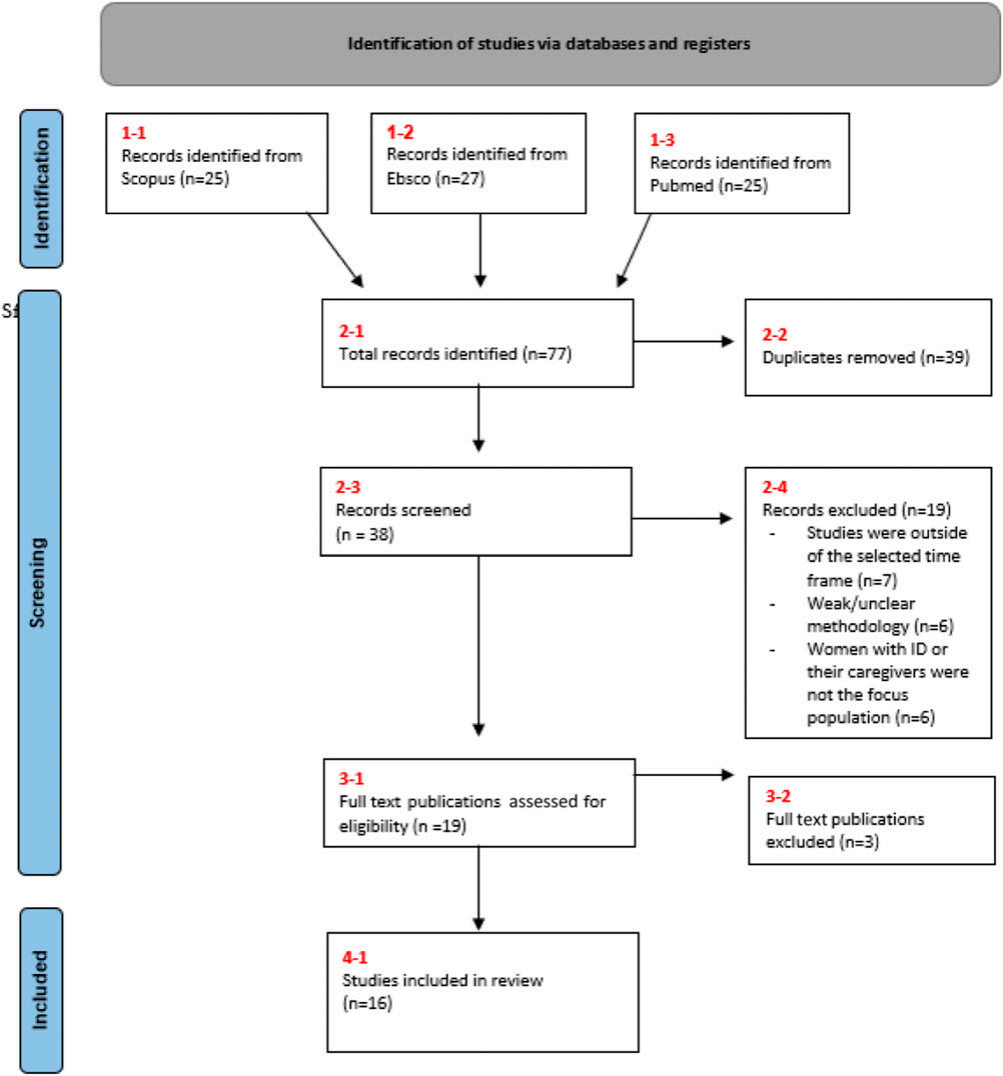
PRISMA flow diagram.

The screening process is diagrammatically represented in a PRISMA-ScR flow diagram (Tricco et al., 2018) in Figure 1.

### Stage 4: Charting the data

A data collection tool was developed to extract the characteristics from each included study and to confirm their relevance to the review objectives. The extraction process took an iterative approach and the tool was revised and adjusted where necessary. The data were collected in a Microsoft Excel spreadsheet for validation and coding. This tool was tested by the research team prior to implementation to ensure that the pertinent information was being captured accurately.

### Stage 5: Collating, synthesising and reporting the results

The abstraction of data was undertaken by one reviewer. To ensure accurate data collection, two additional researchers reviewed and compared the extracted data to ensure consistency and eliminate discrepancies. The data extracted included details about the phenomena of interest (menopause), the population at hand (women with intellectual disabilities and/or their formal and informal caregivers), the study methods and outcomes deemed significant to the review question and specific objectives. The results generated through this review were reported using the PRISMA-ScR tool (Tricco et al., 2018).

The PRISMA-ScR is the most recent and innovative approach for reporting in scoping reviews. The PRISMA-ScR is not a guideline for review synthesis, rather it serves as a checklist tool to guide the comprehensive reporting of methods and findings. As approaches for evidence synthesis in scoping reviews advances, the use of the PRISMA-ScR extension is considered a reliable, practical tool for improving the quality of scoping reviews when used in conjunction with methodological guidance (Peters et al., 2021)

The narrative report synthesises the study findings grounded in themes that emerged from the extracted data. The results are presented in line with the research questions and in the context of the overall scoping review objectives. Table 2 presents summary information on the included articles.

**Table 2.** Overview of each of the 16 sources.

TITLE	Authors	Year	Publication	Context	Methodology	Key findings
*REPRODUCTIVE (IN)JUSTICE AND INEQUALITY IN THE LIVES OF WOMEN WITH INTELLECTUAL DISABILITIES IN SCOTLAND*	Wiseman, P., Ferrie, J.	2020	Scandinavian Journal of Disability Research, 22 (1), pp. 318-329.	Scotland	Open qualitative questionnaire (n=21) and further focus group discussion (3) with 12 women	- Addresses the fact that women with intellectual disabilities are largely absent from mainstream feminist narratives of reproductive justice.
*HEALTHCARE FOR WOMEN WITH DISABILITIES IN THE CLIMACTERIC AND MENOPAUSE*	de Almeida, E.W., Greguol, M.	2015	Sexuality and Disability, 33 (2), pp. 279-298.	Global	Narrative literature review	- Despite many of the women in included studies being overweight or having difficulties in performing daily activities most of the study populations perceived their health as being good.
						- Participants in the studies who were evaluated for level of physical activity were generally classified as being active.
						- Women in some studies did not follow the proposed recommendations for routine health checks.
						- Reported menopausal symptoms from women were not significantly different from those reported by women in the general population.
*MENOPAUSE EXPERIENCES AND ATTITUDES IN WOMEN WITH INTELLECTUAL DISABILITY AND IN THEIR FAMILY CARERS*	Chou, Y.-C., Lu, Z.-Y.J., Pu, C.-Y.	2013	Journal of Intellectual and Developmental Disability, 38 (2), pp. 114-123. Cited 3 times.	Taiwan	Mixed methods study: a survey performed with women with intellectual disabilities (n=7) and their carers (n=65), semi structured interviews with women with intellectual disabilities (n=4) and their carers (n=10).	- Less than one quarter of the women with intellectual disabilities in the survey had received a gynaecological test while a minority had consulted a GP about menopause.
						- Women with intellectual disabilities rarely used HRT and lacked access to regular health check-ups.
						- Almost all women with intellectual disabilities were not informed by their carers about menopausal issues/symptoms.
						- Carers indicated that they did not think it was necessary to prepare women for the menopause.
*CAREGIVER AWARENESS OF REPRODUCTIVE HEALTH ISSUES FOR WOMEN WITH INTELLECTUAL DISABILITIES*	Lin, L.-P., Lin, P.-Y., Hsu, S.-W., Loh, C.-H., Lin, J.-D., Lai, C.-I., Chien, W.-C., Lin, F.-G.	2011	BMC Public Health, 11, art. no. 59, . Cited 14 times.	Welfare institutions in Taiwan.	Cross-sectional, questionnaire-based study (n=1152) from 32 institutions	- Caregivers were familiar with sex education, issues of menopause, and preventive health services but were unfamiliar with issues concerning menstruation in women with intellectual disabilities.
						- Caregivers in intellectual disability services had a clear role in encouraging women to adopt health practices and to ensure that women accessed primary healthcare.
						- Many primary caregivers believed that people with intellectual disabilities lack the capacity to make informed decisions about their sexuality and intimate relationships.
						- Parents and staff differed in their attitudes; parents had more conservative views.
						- Formal caregiver’s gender, educational level, and experience assisting with reproductive health care were significantly associated in logistic regression analyses with high scores for reproductive health awareness for women with intellectual disabilities.
*MENOPAUSAL EXPERIENCES OF WOMEN WITH INTELLECTUAL DISABILITIES*	Willis, D.S., Wishart, J.G., Muir, W.J.	2011	Journal of Applied Research in Intellectual Disabilities, 24 (1), pp. 74-85. Cited 8 times.	Scotland	Semi-structured interviews with 45 women with intellectual disabilities; (17 Down Syndrome, 28 non-Down Syndrome; age 35–65 years).	- Menopausal experiences of the women with intellectual disabilities and those specifically with Down Syndrome were similar.
						- The majority of women were unaware of menopause-associated changes in their body. Few women understood why they had menstruated.
						- It was difficult for women to understand what changes they experienced were as a direct result of the menopause and what symptoms were as a result of other causes.
						- Identified the need for increased training for carers and more tailored resources and health education materials for women with intellectual disabilities.
*CARER KNOWLEDGE AND EXPERIENCES WITH MENOPAUSE IN WOMEN WITH INTELLECTUAL DISABILITIES*	Willis, D.S., Wishart, J.G., Muir, W.J.	2010	Journal of Policy and Practice in Intellectual Disabilities, 7 (1), pp. 42-48. Cited 6 times.	Scotland	Interviews with 69 formal caregivers of 45 women with intellectual disabilities	- Carers reported difficulty separating the psychological and physical consequences of the menopause from behaviours and symptoms arising from other causes.
						- Carers demonstrated a willingness to talk to women with intellectual disabilities about the menopause and symptoms. This was often approached by discussing personal experiences or those of family and friends.
						- Participants were keen to ensure that information they shared was appropriately tailored to the ability level of the individual women they supported.
						- Carers acknowledged the need for health resources to be better suited to needs of women with intellectual disability and for more relevant health education training for staff.
*A DECADE ON: WHAT HAVE WE LEARNT ABOUT SUPPORTING WOMEN WITH INTELLECTUAL DISABILITIES THROUGH THE MENOPAUSE?*	Willis, D.S.	2008	Journal of Intellectual Disabilities, 12 (1), pp. 9-23. Cited 4 times.	UK	Semi-structure interviews with 15 women with intellectual disabilities.	- Women with intellectual disabilities had little understanding or knowledge about the menopause.
						- Women were unable to explain why they had periods and articulated a dislike for and discomfort related to having periods.They expressed feelings of relief and happiness when their periods would stop for good.
						- Women felt it was important to take exercise and eat well but had no apparent understanding of eating specific healthy foods.
						- Some women reported feeling unhappy with their weight or reported that they were on 'diets' indicating a level of autonomy in their decision about their health.
*GOING THROUGH THE MENOPAUSE: PERCEPTIONS AND EXPERIENCES OF WOMEN WITH INTELLECTUAL DISABILITY*	McCarthy, M.	2002	Journal of Intellectual and Developmental Disability, 27 (4), pp. 281-295. Cited 19 times.	UK	Qualitative element of a larger mixed methods study. Here, findings from interviews with 15 women with intellectual disabilities are reported.	- Levels of knowledge about the menopause was generally low.
						- Women’s experiences of menopausal changes were predominantly physical. Some emotional aspects were emphasised, but few psychological and social impacts were reported.
						- Menopausal changes were presented in the context of other broader age-related issues.
						- Most of the women articulated strong preferences for talking only to other women about periods and the menopause.
						- Generally, low levels of choice and autonomy were highlighted.
*RESPONSES TO WOMEN WITH LEARNING DISABILITIES AS THEY GO THROUGH THE MENOPAUSE*	McCarthy, M.	2002	Tizard Learning Disability Review, 7 (1), pp. 4-12. Cited 12 times.	UK	Mixed methods study: semi structured interviews with women with learning disabilities (n=30) and questionnaires with 314 GPs in one health district in South East England.	- Relief from menopausal symptoms for women with intellectual disabilities was reported as being unpredictable.
						- Most GPs lacked comprehensive training to deal with the specific needs of women with intellectual disabilities.
						- The impact of menopause in this population was predominantly reported at the physical level.
						- Women were likely to need carers who were observant and sensitive to facilitate access to health care services.
						- Recommendations suggested it would be beneficial if GPs worked in partnership with the women and their carers.
*WOMEN WITH LEARNING DISABILITIES AND THE MENOPAUSE*	Martin, D.M., Cassidy, G., Ahmad, F., Martin, M.S.	2001	Journal of Learning Disabilities, 5 (2), pp. 121-132. Cited 6 times.	N/A	Narrative review	- Services for people with learning disabilities focused on active sexual health The needs of aging women with intellectual disabilities were often overlooked.
						- The gynaecological issues of (post-menopausal) women with intellectual disabilities were often compounded by anxieties and fears of gynaecologists.
						- Carers of women with intellectual disabilities were more likely to dismiss psychological and physical morbidity among older people with intellectual disability.
						- Appropriate health promotion about menopause for women with intellectual disabilities and their carers was recommended.
*CAREGIVER ATTITUDES TO GYNAECOLOGICAL HEALTH OF WOMEN WITH INTELLECTUAL DISABILITY*	Lin, L.-P., Lin, J.-D., Chu, C.M., Chen, L.-M.	2011	Journal of Intellectual and Developmental Disability, 36 (3), pp. 149-155. Cited 4 times.	Taiwan	Cross-sectional study; structured questionnaire survey with caregivers of women with intellectual disabilities recruited using purposive sampling (n=1152).	- Most respondents considered that they were able to provide appropriate advice on menstrual education for the women with intellectual disabilities under their care.
						- 46% of respondents disagreed or strongly disagreed that the institution should take full responsibility for the provision of education about menstrual issues.
						- 37.8% of respondents considered that hysterectomy was an effective way to deal with severe menstrual problems.
						- Most of the caregivers were in agreement about the implementation of gynaecological healthcare checks
						- 90.1% of the caregivers considered that institutions should limit patients’ freedom in order to avoid sexual problems associated with abandoning contraception use after menopause.
						- 84.7% of respondents disagreed or strongly disagreed that symptom relief was unnecessary, while 97.2% agreed or strongly agreed that a healthy lifestyle was more important.
*CHINESE WOMEN WITH 29-30 CGG REPEATS HAVE AN EARLIER MENOPAUSE*.	Tang, R.; Chen, R.; Luo, M.; Lin, S.; Yu, Q.	2020	Climacteric	Beijing, China	Mixed method. Analysis were part of a prospective, open cohort, community-based longitudinal study, the PALM study, which aimed to investigate ovarian aging in midlife women in China.	- The FMR1 CGG repeat sizes might have played a role in the mechanisms of the ovarian aging process and thereby affected the timing of menopause.
						- Women with 29–30 FMR1 CGG repeats may have experienced menopause approximately 2 years earlier than those carrying 28 or 31 CGG repeats and had a longer FSH fluctuant period.
*DISCUSSING THE MENOPAUSE WITH WOMEN WITH LEARNING DISABILITIES*.	McCarthy M; Millard L	2003	British Journal of Learning Disabilities, 31, 9–17	UK	The findings reported in the present study are from part of large research project, which had four different phases of data collection, all conducted between 1999 and 2000. Presented in this paper is are results from semi structured interviews describing the issues which arise when talking to women with learning disabilities about the menopause.	- Most women did not know or recognize the commonly used terms related to menopause. It was recommended that these terms need to be explained at an early stage and reminders about terminology should be given.
						- The use of pictures and other visual resources was strongly recommended.
						- It was suggested that workers raising issues of ‘how people’s bodies change as they get older’ need to be sensitive to the fact that women may not want to be seen as older and also to the possibility that such discussion may spark memories of bereavement.
						- There appeared to be little role for male staff in directly supporting women with intellectual disabilities.
						- Older female staff who share their experiences can help ‘normalize’ the experience of menopause and sensitivity is needed in discussions of how the menopause effects women.
*FEMALE CARRIERS OF FRAGILE X PREMUTATION HAVE NO INCREASED RISK FOR ADDITIONAL DISEASES OTHER THAN PREMATURE OVARIAN FAILURE.*	Hundscheid RD; Smits AP; Thomas CM; Kiemeney LA; Braat DD	2003	American journal of medical genetics. 117A:6–9 (2003).	Netherlands	Involved. 152 women who were premutation carriers and 112 other women (59 with a normal FMR-1 gene and 53 with a full mutation)	- No statistically significant differences in the occurrence of diseases (such as cardiovascular disease and osteoporosis were found to be associated with menopause.
						- Lower bone mineral density was observed only in women who were premutation carriers but these women were not at increased risk of additional medical problems.
						- Carrying the premutation may have affected a womans ovaries but did not significantly increase the risk for additional medical problems.
*OBESITY ENHANCES VERBAL MEMORY IN POSTMENOPAUSAL WOMEN WITH DOWN SYNDROME.*	Patel BN; Pang D; Stern Y; Silverman W; Kline JK; Mayeux R; Schupf N	2004	Neurobiology of Aging 25 (2004) 159–166	NY, United States	Mixed methods study which: compared the performance of healthy nondemented obese and non-obese women (n=116) with Down Syndrome (DS) on a broad spectrum of cognitive tests and review of clinical assessments.	- Estrone levels were 66.9% higher in obese postmenopausal women than in non-obese postmenopausal women and 136% higher in obese premenopausal women than in non-obese premenopausal women.
						- Obese postmenopausal women performed significantly better than non-obese women on measures of verbal memory and on an omnibus test of neuropsychological function, but did not differ significantly in verbal fluency, language, praxis or visuospatial functioning.
						- Among premenopausal women, there was no difference in cognitive function between obese and non-obese women.
						- Obese postmenopausal women with Down Syndrome performed better than both normal weight and overweight women on tests of verbal explicit memory and performed better than normal weight, but not overweight, women on an omnibus test of neuropsychological functioning.
*ONSET OF DEMENTIA IS ASSOCIATED WITH AGE AT MENOPAUSE IN WOMEN WITH DOWN'S SYNDROME.*	Schupf N; Pang D; Patel BN; Silverman W; Schubert R; Lai F; Kline JK; Stern Y; Ferin M; Tycko B; Mayeux R	2003	Annals of Neurology Vol 54 No 4	NY, United States	Mixed methods. Clinical assessments of postmenopausal women with Down Syndrome, 40 to 60 years of age. Multivariate regression analyses to determine the relation of age at menopause to age at onset of Alzheimer’s disease,	- Women with early onset of menopause (46 years or younger) had earlier onset and increased risk of Alzheimer’s disease (AD) compared with women with onset of menopause after 46 years.
						- Women with dementia had higher mean serum sex hormone binding globulin levels than women without (86.4 vs 56.6 nmol/L, p 0.02), but similar levels of total estradiol, suggesting that bioavailable estradiol, rather than total estradiol, was associated with dementia.

## Results

### Extent of the literature

After the duplicates were removed, 38 published works remained for review. The titles and abstracts were screened against the study inclusion criteria identified and 19 articles were included. Studies that were not relevant to the review research questions were also disqualified. In total, 16 studies were considered relevant for the scoping review. The majority of the studies reviewed were published between 2000 and 2010 (n=9). Five studies were published between 2011 and 2015. The final two articles were published in 2020.

### Characteristics of the literature

Studies were published in journals that reported on key themes of intellectual disability, gender, intellectual, learning and developmental disability, sexual and reproductive health, reproductive rights, sexuality, psychology, and public health. Two studies reported no setting because they were reviews or commentaries. The remaining studies were conducted in Scotland (n =3), the United Kingdom (n =4), Taiwan (n =3) and the United States (n = 2). The Netherlands and China each had one study reviewed. Study participants across the reviewed literature varied. In six of the studies the direct or indirect experiences of women with intellectual disabilities were analysed, two of these studies included women with intellectual disabilities, their informal caregivers, formal care staff and health care staff. One study focused uniquely on the perspectives of informal caregivers of women with intellectual disabilities, and another on the experiences of formal caregivers of women with intellectual disabilities. The informal caregivers of women with intellectual disabilities were predominantly family members, parents and siblings. In the three studies that included formal caregivers and health care staff, these participants were paid staff from residential and day care settings and general practitioners. Studies used varying terminology to describe intellectual disability however the overall classification of intellectual disability appeared consistent.^1^ Two studies included women with Down Syndrome uniquely. One study from the Netherlands involved female carriers of fragile X^2^ (Hundscheid et al., 2003) and another from China focused on 29-30 CGG repeats (Tang et al., 2020).^3^

Of the reviewed research, 75% (n=12) of the studies were qualitative (n=6) and mixed methods (n=6) in design. Two studies (12.5%) employed a quantitative research approach and two publications (12.5%) were reviews or commentaries. Qualitative and mixed methods studies that included women with intellectual disabilities employed semi-structured interview and focus groups discussion methods. One study also used pictorial prompts to assist the women. One qualitative study included a qualitative questionnaire for women with intellectual disabilities in addition to focus group discussions. Another administered a survey to women and their caregivers and was followed-up with semi structured interviews. Mixed methods studies included a broad range of qualitative and quantitative methods including interviews, observations, surveys, questionnaires, reviews of clinical notes and neurological examinations. Two of the studies were longitudinal in design, two included a review of medical records and a further two included clinical assessment of participants. Of the two quantitative research studies, both employed survey methods and were cross-sectional in design. The analysis of the literature identified six primary themes relating to the menopausal experiences of women with intellectual disabilities.

### Perceptions of general health and healthy living

Keeping healthy after the menopause is important for all women as at the age of menopause women are at an increased risk of cardiovascular disease osteoporosis and cancer (Willis et al., 2011). However, the menopausal symptoms and bodily changes that occur during menopause can reduce feelings of vitality and make keeping fit and healthy more challenging (Deeks and Mccabe, 2004). Three of the studies in this review discussed the general health and perceptions of health amongst women with intellectual disabilities and their caregivers. Willis et al., 2011 found that 43 of the 45 women interviewed described themselves as being in good health. Two of the women who had described themselves as healthy also indicated that they had high blood pressure, and another said she often took fits. Similarly, an earlier study by Willis et al., 2011 with carers of women with intellectual disability, suggested that the majority of those interviewed reported the health of the woman with intellectual disabilities in their care to be ‘good’. Levels of knowledge of what ‘good’ health represented was not clear. These findings were consistent with data from the IDS-TILDA longitudinal study on ageing in Ireland among people with an intellectual disability aged 40 and over. In this Irish study participants reported being in good health despite an increase in diagnosis of chronic health conditions such as cardiac issues, epilepsy, constipation, arthritis, osteoporosis, urinary incontinence, falls, cancer, and thyroid disease (Mccarron et al., 2017). All post-menopausal women are at an increased risk of osteoporosis. However, women with intellectual disabilities are at a higher risk of developing osteoporosis (Mccarthy, 2002a). Therefore, maintaining a calcium rich diet is important for the prevention of osteoporosis especially in women with intellectual disabilities. Despite this, Mccarthy, 2002a found that women knew little about how to protect themselves from osteoporosis and only two women of the 30 women interviewed appeared to know a food which contained calcium.

### Knowledge of menstruation and menopause

Two of the articles considered in this review identified the paucity of social research focusing on the menstrual or menopausal experiences of women and girls with disabilities (Mccarthy, 2002a; Mccarthy and Millard, 2003) and comparable reproductive health information. Eight (50%, 8/16) studies highlighted the low levels of knowledge that women with intellectual disabilities displayed about reproductive health in a general sense. This was attributed to the global ‘legacy of inequality’ which had been put upon disabled women, particularly women with intellectual disabilities’(Wiseman and Ferrie, 2020) and exacerbated by a research agenda which frequently reflected the needs of the researchers rather than the needs of women living with intellectual disabilities themselves (Gilbert, 2004).

In the nine articles that discussed menstruation, the majority of women involved in the studies were unable to confirm when their periods began (or relate their menstrual cycle to their ability to have children) (Willis et al., 2011). Instead, many women reported the physical discomfort and pain associated with having their periods (Willis et al., 2011). Articles that included women with intellectual disabilities in their study population concluded that knowledge of menopause was equally limited. Most women did not know or recognise the commonly used terms for referring to menopause or menopausal transition and in many cases the research team needed to explain and provide reminders of the terminology when conducting data collection with women with intellectual disabilities. In addition, lack of knowledge amongst women with intellectual disabilities about menopause was found to account for why some of the women experienced physical side effects of the menopause but were unable to identify the symptoms (Willis, 2008).

### Menopausal symptoms and bodily changes

Nine (53%) of studies discussed the negative effects menopausal transition on women’s health. When asked about bodily changes because of getting older, many women reported no changes at all, or focused primarily on physical ailments (Willis, 2008), few women noticed specific symptoms as a result of menopause itself. When prompted by researchers, in six studies with women with intellectual disabilities, ‘hot flushes’ emerged as the most recognised symptom. Additional symptoms such as cold feed and hands (Almeida and Greguol, 2015) weight gain, stomach pain, hair thinning, and growth of facial hair (Mccarthy, 2002a) were noted in only two studies. Of the changes that were reported it could not be determined whether the origins of these symptoms were as a result of menopause (Willis, 2008) or were characteristics of the disability, or the aging process itself (Almeida and Greguol, 2015). Chou et al., 2013 found that family carers of women in peri- or post-menopause were more likely to recognise symptoms than those caring for pre-menopausal women, attributable perhaps to women having milder symptoms, or carers not having observed significant changes in their physical or psychological wellbeing. GPs reported three primary reasons for which women with intellectual disabilities presented with medical concerns related to the menopause, these included hot flushes, problems with menstruation (such as heavy or irregular bleeding), changes in mood or behaviour (Mccarthy, 2002b).

### Women’s access to health care

Access and utilisation of health care is identified as a key health indicator for people with intellectual disability (Ouellette-Kuntz, 2005). Irish research suggests that use of GP services is marginally higher amongst people with intellectual disabilities than in the general population (95.8% and 91% respectively) and that increased use of services is generally consistent with aging (Mccarron et al., 2017). In contrast, a study exploring the scope for women with intellectual disabilities to exercise choice and control over access to contraception, revealed that in accessing reproductive health care, women with intellectual were often not given adequate time, sensitivity, or accessible information by health professionals to make decisions (Mccarthy, 2009). For some women access to health care was fraught with tensions around their bodily autonomy and having their lives controlled by others. One woman with intellectual disabilities interviewed by Mccarthy and Millard, 2003 complained that while she wanted to look after her own health and when seeking to access healthcare she was never given privacy or authorisation to do so. She articulated fustration with the fact that she was always accompanied by are carer, and noted ‘we’ve got to have staff all the time’(Mccarthy and Millard, 2003). This was supported by findings from Mccarthy, 2002b in which 89% of GPs surveyed reported that the women with intellectual disabilities always or generally came to seek care with a caregiver. Some GPs in this study acknowledged that in some cases this could be regarded positively, as caregivers can be strong advocates for women in obtaining health care. Other respondents however, recognised that being chaperoned by a caregiver could also have negative consequences particularly if women perceived that these caregivers (both formal and informal) were questioning their intentions for requesting to see a doctor (e.g. inappropriately seeking attention), ‘they don’t give me permission, in case I’m messing about deliberately or something’ (Mccarthy, 2002b).

A high level of caregiver involvement in health seeking and symptom management was discussed in one study involving caregivers of women with intellectual disabilities. It was noted that mothers of pre-menopausal women anticipated needing a high level of medical intervention over the course of their daughter’s menopausal transition and some mothers (n=7) predicted that they would seek out the support of a GP (Mccarthy, 2002b). However, amongst mothers of peri- and post-menopausal women (n=8), half had not seen a doctor, two had received specific medical treatment for menopause (HRT) and one mother had to request tailored information about her daughters’ diet during menopause (Mccarthy, 2002b) suggesting that levels of intervention needed during transition were lower than may have been expected. It appeared that women with intellectual disabilities frequently lacked control and decision making with regards to their medical care during menopause and these decisions were reported to fall to their caregivers (both informal and formal).

Laws related to the involuntary sterilisation of women with intellectual disability vary and although dated, Lin et al., 2011a, found in interviews with parents and caregivers of women with intellectual disabilities, that despite the ban on involuntary sterilization, many parents and caregivers would support sterilization as a form of contraception, especially for women with severe intellectual disabilities. Contemporary findings from research conducted with medical doctors in Australia identified a high level of incongruity with the legislation prohibiting sterilisation for individuals with intellectual disabilities, highlighting concerns for the broader context of human rights for people with disabilities (Gilmore and Malcolm, 2014). An additional study also conducted by Lin et al., 2011b also found that 90.1% of the caregivers interviewed in Taiwan believed that institutions should limit the freedom of women with intellectual disabilities in order to avoid sexual ‘problems ‘associated with lack of contraceptive use after menopause.

Inadequate access to routine reproductive health care services including breast cancer screenings and Pap Smear screenings were discussed. The tendency of health professionals to direct questions to caregivers and family members rather than to the women with intellectual disabilities themselves only furthered the denial of women’s agency and as a result, women with intellectual disabilities were subject to taking medical direction and had made decisions based on informed compliance rather than informed choice (Wiseman and Ferrie, 2020). While it was acknowledged that women with intellectual disabilities may find decision making – particularly in relation to HRT – a complex task, GPs reported variation in attitudes and practices towards consent in this population. Mccarthy, 2002b reported that only five GPs (of n = 73) mentioned issues relating to consent and treatment, one suggesting that ‘modern/good practice dictates that the pros and cons of HRT (for example) are discussed (i.e. the patient is informed and the patient decides after considering the options)’(Mccarthy, 2002b).

### Sources of knowledge information and support

Of the studies reviewed, sources of information and support for women with intellectual disabilities experiencing menopause varied greatly. Mccarthy, 2002a reported that amongst the 15 women with intellectual disabilities interviewed six women said they had never discussed menopause with anyone, three women said they had spoken to doctors, four to staff in the services and two to their mothers. Similarly, in Willis et al., 2011 qualitative study with 45 women with intellectual disabilities under two thirds of participants said that they had reached out to someone for support during menopausal transition and of those do did, key workers, doctors, family and friends were found to be the most reliable sources of information.

Learning from female staff in residential and day services was a clear choice of most of the women in this study, with the exception of those who still lived at home with their mothers. Formal caregivers in the UK recognised the importance of their role in providing information about the menopause to women with intellectual disabilities, yet caregivers who were themselves menopausal or post-menopausal, were observed to be embarrassed and reluctant to discuss the topic (Mccarthy and Millard, 2003). In one study, respondents (caregivers working in Taiwanese government run day-care and residential institutions) considered that they were able to provide appropriate advice about the menopause, however 46% of the study population either disagreed or strongly disagreed that the institution should take full responsibility for the provision of education about these issues (Lin et al., 2011b). Male GPs were often cited as barriers to information about health issues due to gendered power dynamics where women felt embarrassed or worried about talking to a man, especially men who were felt to be in a position of authority (Wiseman and Ferrie, 2020). Three studies indicated the importance of female support for women in discussions of reproductive health, with women emphasising a preference to speak to another female about what they considered to be ‘women’s problems’ (Chou et al., 2013; Mccarthy and Millard, 2003; Willis et al., 2011). Of 11 male formal care staff (of a population of 66) who responded to a survey four did not think their gender caused difficulties in supporting women through the menopause (Mccarthy, 2002b).

In discussions about sexual and reproductive health in a general sense, Scottish women reported that parents, particularly mothers were important sources of health education (Wiseman and Ferrie, 2020). Similarly, other studies from the UK revealed that relatives were very well placed to explain the menopause to women with intellectual disabilities. This was only relevant in cases where women still resided in the home with parents and siblings (Mccarthy, 2002b). Women with intellectual disabilities living alone with male relatives were at a disadvantage in accessing information about menopause as there were less opportunities for discussion of ‘women’s problems’ (Mccarthy and Millard, 2003).

However, Wiseman and Ferrie, 2020 reported that lack of information amongst older women with intellectual disabilities about menopause could be linked to information being withheld by parents and that amongst older women with intellectual disabilities, parents and families frequently acted as gatekeepers to women’s bodily autonomy. Attitudes of parents and staff differed slightly, and parents were considered to take a more conservative approach to the information they provided to women than formal caregiver and healthcare professionals (Lin et al., 2011a). Parents of older women with intellectual disability and who were gatekeepers for their bodily autonomy, reportedly percieved that having an intellectual disability was in direct conflict with informed sexual citizenship and this manifested in them withholding information, the spread of misinformation, and difficult relationships with heath care professionals (Wiseman and Ferrie, 2020). Mccarthy, 2002b acknowledged that complex parent-heath professional relationships emerged for parents of older women as it was more difficult for them to accept advice from professionals about the menopause for their daughters particularly if the medical ideas or theories were more liberal than their own.

Other important and useful sources of information and health education reported by women included sharing experiences and discussions through women’s groups (Mccarthy and Millard, 2003), and through TV programmes and soaps showcasing women’s experiences of menopausal transition (Mccarthy and Millard, 2003; Willis, 2008). One study emphasised how specialist resources could support women in discussions about menopause, validating ideas though pictures that were sensitive to issues that women may otherwise find it difficult to discuss (Mccarthy and Millard, 2003).

### Knowledge and attitudes of caregivers and health professionals

Although women reached out to formal and informal caregivers and health professionals, it was clear that those in caregiving roles were not always themselves aware of women’s needs during menopause. In one study in Taiwan, a linear regression indicated that carer age and education, but not carer gender, were associated with their self-reported attitudes (assessed using the Menopause Attitude) towards post-menopausal women (Chou et al., 2013). In a review of caregiver awareness of reproductive health issues for women with intellectual disabilities, Lin et al., 2011a concluded that caregivers were familiar with sex education, issues of menopause, and preventive health services (mean score≧4), but they were unfamiliar with these issues in the context of women with intellectual disabilities. Of the two studies which included GPs in the UK (Mccarthy, 2002b; Mccarthy and Millard, 2003), only one presented GPs knowledge and perceptions of menopause in women with intellectual disabilities. The article reported that several doctors involved in the research reflected that the menopause was not an issue which they had seriously considered in relation to women with intellectual disabilities and a number of those involved concluded that they should proactively reach out to women with intellectual disabilities and/or their carers. In contrast however, others believed that there was no need to engage women in relation to the menopause, with one GP suggesting that menopause was of “low significance” to women in this population (Mccarthy, 2002b).

## Discussion

This paper reviews the literature published in the last 20 years which examines the menopausal experiences of women with intellectual disabilities. As discussed, research in the area of menopause is scarce and of the studies that do exist, including many in this review, findings have primarily focused on the symptoms of menopause with limited research documenting how menopause affects women themselves through their lived experiences. Further, dated research in this area has long term repercussions for the identification and management of menopausal symptoms of women with intellectual disabilities.

Adults who lack sexual information may feel embarrassed discussing issues around reproductive health and menopause. It is important therefore to approach education about the topic in a sensitive manner where autonomy and decision making are promoted in an environment where it is safe to ask questions and women’s voices and experiences are heard. Some women may prefer to discuss their experiences or concerns without family members or caregivers present and women’s autonomy should be safeguarded in as much as is possible. Women who are their own legal guardians have the right to make medical decisions for themselves, including decisions about reproductive healthcare. If a patient has a legal guardian, the guardian must consent to medical decisions, but it may still be appropriate to provide opportunities for private conversation during medical visits. It is important therefore to approach education and information about symptoms and treatment in a sensitive manner, in an environment where it is safe to ask questions and where autonomy and decision making are encouraged.

### Considerations for future research

The review has identified that the provision of information and education for women with intellectual disabilities is limited. Further research is needed on the actual experiences of women which is inclusive and tailored to the cogitative needs of the women themselves. Research is also needed to understand caregiver and health care providers needs in providing health education and related to best practices for supporting women with intellectual disabilities through menopause in terms of information sharing, symptom management and treatment, and health screening. The UN Convention on the rights of people with disabilities outlines the right of people with disabilities to receive information about reproductive health and family planning and the right to receive comprehensive healthcare services without disability-based discrimination (UN General Assembly, Assembly, 2007). Further enquiry is needed to investigate if women of menopausal age can access these rights.

### Implication for carers and health care providers

This review indicates the need for better awareness of menopause and menopausal needs of women with intellectual disabilities. Historically, there has been little attention paid to the specific and distinct reproductive needs of this population. Women who are currently experiencing menopausal transition were likely growing up in the 1970s and 1980’s when reproductive health, menstruation and menopause were not openly discussed. Women with intellectual disabilities, who may not have understood menstruation in the first place, are now faced with trying to understand the changes brought about my menopause.

The need for improved education of formal and informal carers and healthcare providers, and improved health screening better support women through menopausal transition is evident. Health care providers should be equipped to deliver tailored and appropriate reproductive health education and treatment when it is relevant and feasible. It should be acknowledged that family members may have strong opinions about the needs of their loved ones as they reach the age of onset of menopause. Health care providers should be encouraged to individualize care and promote women’s autonomy, meanwhile recognising that it may be appropriate to include family members or key caregivers in patient education regarding menopause and appropriate treatments. Evidence suggests that exposure to people with intellectual disabilities increases provider comfort in caring for this population (Mann et al., 2022). Structures should be put in place to enable healthcare providers and formal carers to seek education and opportunities for exposure to adults with intellectual disabilities and greater consideration of health issues specific to women with intellectual disabilities is needed inmedical education. Further these groups should seek to familiarize themselves with the barrier’s women with intellectual disabilities face in accessing information, treatment, and care for menopause related issues.

## Conclusion

The findings from this review reveal the complex issues women with intellectual disabilities face related to their sexual and reproductive health and how their previous experiences influence their knowledge and perceptions of menopausal transition. The paucity of studies explicitly investigating the lived experiences of women with through menopause provides justification of the need for greater investment in future research to guide policy and practice in this area. Finally, specific guidance is needed for the care of women with intellectual disabilities at the age of onset of menopause, which considers their complex physical, psychological, communication and health-seeking behaviour needs.

## Notes

1. A person is considered to have an intellectual disability when their general intellectual functioning is significantly below average; significant deficits exist in adaptive skills and the condition originates from childhood before the age of 22).

2. Fragile X syndrome (FXS) is a genetic disorder which is caused by changes in a gene called Fragile X messenger called Ribonucleoprotein 1 (FMR1). Symptoms of FXS can include developmental delays, intellectual disabilities, and social and behavior issues.

3. At the beginning of the FMR1 gene there is a repeated sequence of chemical bases, known by the letters CGG. The length of the CGG repeated sequence contributes to the variation in age at onset of menopause.
